# Stress-Induced Production of Bioactive Oxylipins in Marine Microalgae

**DOI:** 10.3390/md22090406

**Published:** 2024-09-04

**Authors:** Amandyne Linares-Maurizi, Rana Awad, Anaelle Durbec, Guillaume Reversat, Valérie Gros, Jean-Marie Galano, Justine Bertrand-Michel, Thierry Durand, Rémi Pradelles, Camille Oger, Claire Vigor

**Affiliations:** 1 Institut des Biomolécules Max Mousseron, IBMM, Université de Montpellier, CNRS, ENSCM, 34093 Montpellier, Francerana.awad@umontpellier.fr (R.A.); guillaume.reversat@umontpellier.fr (G.R.); valerie.gros@umontpellier.fr (V.G.); jean-marie.galano@umontpellier.fr (J.-M.G.); thierry.durand@umontpellier.fr (T.D.); camille.oger@umontpellier.fr (C.O.); 2 Microphyt, 713 Route de Mudaison, 34670 Baillargues, France; remi.pradelles@microphyt.eu; 3 MetaToul, MetaboHUB, Inserm/UPS UMR 1048, I2MC, Institut des Maladies Métaboliques et Cardiovasculaires, 31077 Toulouse, France; anaelle.durbec@inserm.fr (A.D.); justine.bertrand-michel@inserm.fr (J.B.-M.)

**Keywords:** microalgae, oxidative stress, osmotic stress, oxylipins, LC-MS/MS

## Abstract

Microalgae, stemming from a complex evolutionary lineage, possess a metabolic composition influenced by their evolutionary journey. They have the capacity to generate diverse polyunsaturated fatty acids (PUFAs), akin to those found in terrestrial plants and oily fish. Also, because of their numerous double bonds, these metabolic compounds are prone to oxidation processes, leading to the creation of valuable bioactive molecules called oxylipins. Moreover, owing to their adaptability across various environments, microalgae offer an intriguing avenue for biosynthesizing these compounds. Thus, modifying the culture conditions could potentially impact the profiles of oxylipins. Indeed, the accumulation of oxylipins in microalgae is subject to the influence of growth conditions, nutrient availability, and stressors, and adjusting these factors can enhance their production in microalgae culture. Consequently, the present study scrutinized the LC-MS/MS profiles of oxylipins from three marine microalgae species (two Haptagophytes and one Chlorophyte) cultivated in 1 L of photobioreactors under varying stress-inducing conditions, such as the introduction of H_2_O_2_, EtOAc, and NaCl, during their exponential growth phase. Approximately 50 oxylipins were identified, exhibiting different concentrations depending on the species and growth circumstances. This research suggests that microalgae metabolisms can be steered toward the production of bioactive oxylipins through modifications in the culture conditions. In this instance, the application of a low dose of hydrogen peroxide to Mi 124 appears to stimulate the production of nonenzymatic oxylipins. For Mi136, it is the application of salt stress that seems to increase the overall production of oxylipins. In the case of Mi 168, either a low concentration of H_2_O_2_ or a high concentration of AcOEt appears to have this effect.

## 1. Introduction

Microalgae, known for their remarkable diversity, are photosynthetic microorganisms found thriving in a wide array of habitats, ranging from freshwater to marine ecosystems. Their impact is profound, as they contribute to approximately half of the world’s atmospheric oxygen. Positioned at the core of carbon and biochemical cycles, they wield influence in both freshwater and saltwater environments [1]. Moreover, microalgae play a crucial role as a primary food source in aquatic ecosystems, showcasing remarkable cellular adaptability [2].

Microalgae have evolved various adaptation mechanisms to navigate environmental fluctuations, including changes in light intensity, temperature, salinity, pH, nutrient availability, and oxidative stress [3,4]. These adaptations empower microalgae not only to endure but also to flourish in a wide array of often demanding environmental conditions. Leveraging large-scale cultures, microalgae cultivation can yield substantial biomass production [4,5,6]. Photobioreactors, in particular, offer a promising avenue for industrial-scale production, primarily focusing on augmenting algal biomass because of their rapid reproduction rates and other advantageous features [5,6].

This biomass serves as a valuable reservoir of natural bioactive compounds, including long-chain polyunsaturated fatty acids (PUFAs). Depending on the species of algae, various PUFAs can be synthesized in differing amounts. While omega-6 PUFAs may be less prevalent in microalgae compared to macroalgae, certain species may contain linoleic acid (LA; C18:2n−6), arachidonic acid (ARA; C20:4n−6), or docosapentaenoic acid (DPA; C22:5n-6). Among microalgae, three main omega-3 PUFAs are typically identified: α-linolenic acid (ALA; C18:3n-3), eicosapentaenoic acid (EPA; C20: 5n−3), and docosahexaenoic acid (DHA; C22:6n−3). Notably, while both land plants and microalgae can produce ALA, it is only the latter organisms that possess the enzymatic capacity to convert ALA into EPA and DHA [3,7,8,9].

**Figure 1:**
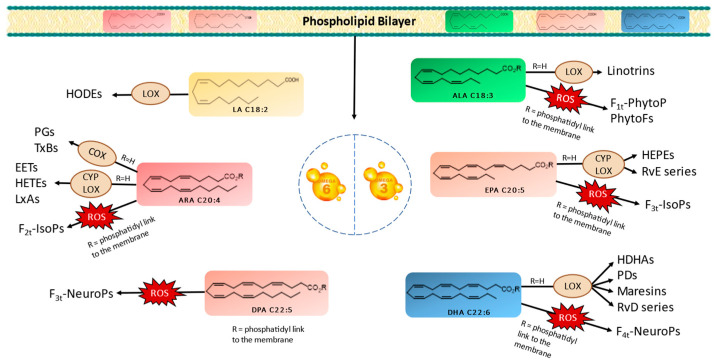
Enzymatic and nonenzymatic pathways of omega-3 and omega-6 PUFAs and their oxygenated derivatives in marine microalgae. Linoleic acid (LA), alpha-linolenic acid (ALA), arachidonic acid (ARA), eicosapentaenoic acid (EPA), docosapentanoic acid (DPA), docosahexaenoic acid (DHA), lipoxygenase (LOX), cytochrome (CYP), cyclooxygenase (COX), hydroxyoctadecadienoic acids (HODEs), prostagladines (PGs), thromboxanes (TxBs), epoxyeicosatrienoic acids (EETs), hydroxyeicosatetraenoic acids (HETEs), lipoxins A (LxAs), hydroxyeicosapentaenoic acid (HEPEs), resolvins Es (RvE), resolvins Ds (RvD), hydroxydocosahexaenoic acid (HDHA), protectins Ds (PDs), phytoprostanes (PhytoPs), phytofurans (PhytoFs), isoprostanes (IsoPs), and neuroprostanes (NeuroP).

PUFAs serve as crucial structural components of cell membranes. However, owing to the presence of carbon–carbon double bonds forming bis-allylic systems, PUFAs are highly susceptible to peroxidation. This oxidative process results in the generation of oxygenated fatty acid metabolites known as oxylipins. It is worth noting that two biosynthetic pathways exist in vivo for oxylipin production. Oxylipins can be generated either within enzyme catalytic sites or within the lipidic membrane itself. These pathways are categorized as enzymatic or nonenzymatic biosynthesis, respectively [10,11,12,13]. Figure 1 provides a nonexhaustive representation of oxylipins formed through enzymatic or nonenzymatic processes, originating from various parent PUFAs. Importantly, oxylipins play significant roles in a wide array of physiological and pathological processes, including inflammation, pain, fever, blood clotting, and cancer [11,14].

The enzymatic oxidation of PUFAs begins with their liberation from the membrane, which is facilitated by phospholipase A2 (PLA2) [11,12,13,15]. Subsequently, specific enzymes, such as cyclooxygenases (COXs), lipoxygenases (LOXs), and cytochrome P450s (CYPs), catalyze the conversion of PUFAs into various oxylipin types, including prostaglandins (PGs), thromboxanes (TxBs), monohydroxylated (e.g., hydroxyeicosatetraenoic acids/HETEs and hydroxyeicosapentaenoic acids/HEPEs), or dihydroxylated oxylipins (e.g., linotrins), epoxides (e.g., epoxyeicosatrienoic acids/EETs), and other complex structures, such as maresins, resolvins, and protectins [13,16]. It is noteworthy that LOX serves as the primary enzyme responsible for generating enzymatic oxylipins in plants, mosses, fungi, and eukaryotic algae [13,14,16].

In contrast, the nonenzymatic oxidation of PUFAs occurs during oxidative stress conditions via reactive oxygen species (ROS), like superoxide and hydrogen peroxide (H_2_O_2_). These radicals react with PUFAs in the presence of molecular oxygen, inside the bilayer membrane, yielding oxylipin structures distinct from those formed enzymatically. Primarily, isoprostanoids, such as phytoprostanes (PhytoPs), isoprostanes (IsoPs), neuroprostanes (NeuroPs), or isofuranoids (IsoFs) are produced [11,13]. At this stage, the oxylipins within the bilayer are released with the involvement of phospholipases.

The biosynthesis of oxylipins is a highly intricate process wherein both enzymatic and nonenzymatic pathways coexist, sharing common biosynthetic intermediates. Moreover, the production of oxylipins in microalgae is subject to influence by various factors, including growth conditions, nutrient availability, and stressors. Under stressful conditions, such as nutrient deprivation or exposure to high light levels, microalgae can elevate the production of oxylipins as a stress response. Several stress conditions have already been investigated, including the use of phytohormones [17,18,19], as well as the alteration of culture parameters inducing abiotic stress [20,21,22], such as nutritional deficiencies [23,24,25,26,27], saline conditions [28,29,30,31], and exposure to chemical stressors [32,33,34,35,36].

Inducing oxidative, nutritional, and saline stresses emerges as a promising strategy to enhance the accumulation of oxidized metabolites by microalgae, particularly for scalable industrial applications. Notably, oxidative stress can be induced through the addition of high levels of ROS, notably H_2_O_2_. Previous studies have shown that the supplementation with 0.5 mM [34], 0.25 mM, 0.75 mM [32], and 1 mM [37] H_2_O_2_ results in increased oxylipin production.

In addition, microalgae cultivated in a medium containing ethyl acetate (EtOAc) can utilize this organic compound as a carbon source, accelerating the carbon flux toward fatty acid synthesis [24,38]. However, this metabolic process may generate ROS as byproducts, contributing to oxidative stress [39]. EtOAc, commonly employed in the biofuels industry, redirects microalgae metabolism toward lipid synthesis, preserving energy reserves crucial for long-term survival [28,40,41]. This stress condition also disrupts cellular ionic balance, leading to excessive accumulation of calcium ions (Ca^2+^) and sodium ions (Na^+^) while reducing potassium ions (K^+^). This ionic imbalance can further enhance lipid production [23] and activate enzymes like PLA2, facilitating the release of lipids into the intracellular space. Consequently, these lipids come into contact with enzymatic machinery, ultimately leading to increased lipid peroxidation [42].

The aim of this study is to leverage the significant cellular adaptability of microalgae and, thereby, steer their metabolism toward enhanced production of optimal oxylipins by adjusting culture conditions. Our objective is to achieve a balance between oxylipin accumulation and microalgae growth, recognizing that certain stressors may hinder the latter.

Building upon our prior research [6], which analyzed the enzymatic and nonenzymatic metabolites of PUFAs across multiple microalgae species, the following three were selected based on their notable abundance of DHA and/or EPA derivatives: two Haptagophytes species (Mi124 and Mi136) and one Chlorophyte species (Mi168). Additionally, our previous work demonstrated the capacity of various PUFAs, which are major constituents of each microalga, to undergo conversion into oxylipins.

We previously observed that Mi124, with its high DHA content, predominantly generates oxidized derivatives of DHA. Conversely, Mi136, characterized by a high profile of EPA, produces abundant oxylipins derived from DHA. Mi168, rich in both EPA and DHA, yields oxidized metabolites of both PUFAs. Numerous studies have investigated the PUFAs abundance in these microalgae species, some of which have analyzed their oxygenated metabolites or explored the influence of stress conditions on oxylipin production rates [43,44,45,46,47,48,49].

In this study, the selected microalgae species were subjected to various stress conditions, including oxidative, carbon, and oxidative stresses, at different concentrations to assess their impact on oxylipin production. It is important to note that the concentrations of the stressors varied for each species to maximize cell viability while inducing a measurable impact on the culture. Additionally, to comprehensively analyze the oxylipin profiles of these microalgae, metabolites of numerous PUFAs, beyond those derived from DHA or EPA, were examined.

## 2. Results

In the present study, we investigated the enzymatic and nonenzymatic oxylipin profiles of three distinct marine microalgae species (Mi124, Mi136, and Mi168) cultivated under diverse stress conditions, including H_2_O_2_, EtOAc, and sodium chloride (NaCl), at two different dosage levels (low and high), when cell survival was not affected, as outlined in the Section 4 (Materials and Methods).

### 2.1. Profiles of Enzymatic and Nonenzymatic Oxygenated Metabolites in Stressed Mi124

**Figure 2:**
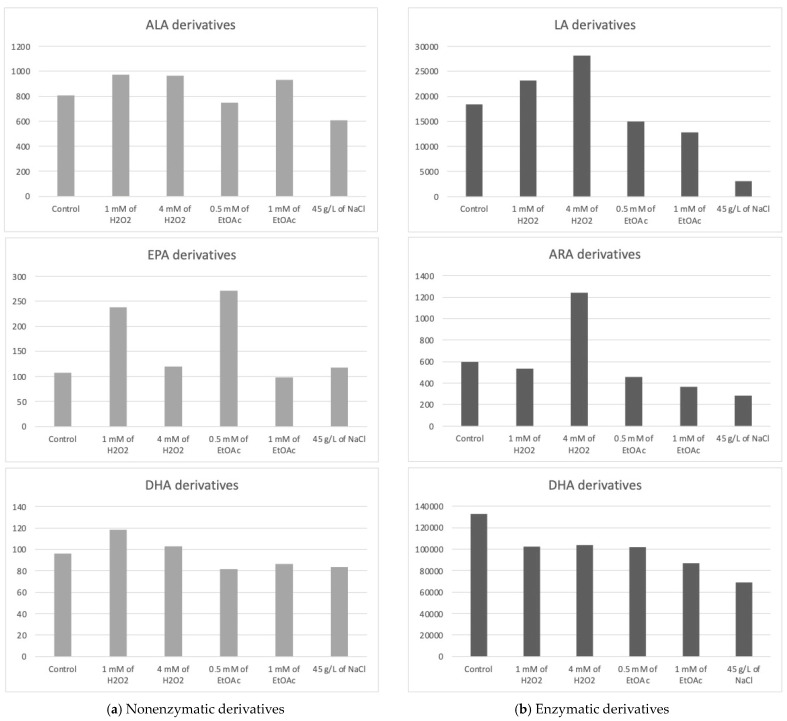
Sums of the concentrations, expressed in pg/mg DWB, of oxygenated metabolites classified by PUFAs in Mi124 depending on growing conditions: control condition versus stress-inducing conditions (H_2_O_2_ at 1 mM or 4 mM; EtOAc at 0.5 or 1 mM; and NaCl at 45 g/L).

The Haptophyte species “Mi124” exhibited some characteristics, particularly its high contents of ALA and DHA. Previous studies highlighted this species’ unique profile [6,46,47]. Upon analysis of its oxylipin profile, we identified the presence of 21 nonenzymatic and 15 enzymatic oxylipins [6]. The concentrations of these metabolites varied slightly depending on the culturing conditions employed. The following table (Table 1) lists the measured concentrations of each metabolite under the different culture conditions. The subsequent figure (Figure 2) represents the total concentrations of enzymatic and nonenzymatic metabolites, respectively, based on the initial PUFA. The graphs provide a clearer view of the trends in metabolite production in response to the applied stress conditions. It is important to note that these are merely trends, as the high variability observed for each condition does not allow for significant differences in most cas. 

#### 2.1.1. Profiles of Nonenzymatic Oxygenated Metabolites

##### Derivatives from ARA (C20:4n−6) and DPA (C22:5n−6)

Among the oxylipins originating from omega-6 PUFAs, the following two specific metabolites were detected: 15-A_2_-IsoP (from ARA) and 4(RS)-4-F_3t_-NeuroP (from DPA n-6).

The addition of 1 mM of H_2_O_2_ would potentially lead to an apparent increase in the concentration of 15-A_2_-IsoP. However, it should be noted that these observations only indicate trends, as the substantial variation among replicates results in a lack of statistical significance.

Similarly, the derivative resulting from the oxidation of DPA, 4(RS)-4-F3t-NeuroP, also appears to be affected by the application of abiotic stress, this time induced by the addition of a low dose of H_2_O_2_ (1 mM). Indeed, we observed an almost four-fold increase in the content of this metabolite in Mi124. However, these findings should be interpreted cautiously, as they reflect trends rather than statistically significant results due to the high variability among replicates.

##### Derivatives from ALA (C18:3n−3)

The derivatives of ALA nonenzymatic oxidations are named phytoprostanes (PhytoPs) and phytofuranes (PhytoFs). In Mi124 species, five PhytoPs and six PhytoFs were quantified.

The total concentration of PhytoPs and PhytoFs exhibited low variations, ranging from 609 pg/mg DWB under saline stress (45 g/L of NaCl) to 964 pg/mg DWB under low oxidative stress conditions (1 mM of H_2_O_2_) (Figure 2a). Specifically, the concentration of 9-F_1t_-PhytoP, 9-L_1t_-PhytoP, and 16-B_1t_-PhytoP seemed to be increasing slightly under H_2_O_2_ stress regardless of the applied concentration, while 16-F_1t_-IsoP and its epimer seem to increase under both H_2_O_2_ and EtOAc stresses.

The total concentration of PhytoFs might be notably higher in the presence of 1 mM of EtOAc or H_2_O_2_. In contrast, the overall content is lower under saline culture conditions (45 g/L).

##### Derivatives from EPA (C20:5n−3)

Under all culture conditions, only three EPA derivatives were detectable, the 5(*R*)-F_3t_-IsoP, 8(*R*)-F_3t_-IsoP, and 18(*R*)-F_3t_-IsoP. Among the EPA derivatives detected, 8(*R*)-F_3t_-IsoP was found in lower concentrations compared to the other derivatives. Once again, the excessive variability in the results obtained prevented us from definitively establishing the optimal conditions for producing oxylipins derived from EPA in substantial quantities. However, these data suggest a beneficial effect of EtOAc (0.5 mM) in their synthesis. Conversely, a high dose of EtOAc would have a deleterious effect.

##### Derivatives from DHA (C22:6n−3)

Five nonenzymatic oxylipins derived from DHA were highlighted in all culture conditions. Once again, we were faced with excessive variability in the results, making it impossible to draw clear conclusions.

Under oxidative stress conditions with the addition of H_2_O_2_, the concentration of 4(*RS*)-4-F_4t_-NeuroP seemed to increase, as well as those of 13A(*RS*)-13-F_4t_-NeuroP and 13B(*RS*)-13-F_4t_-NeuroP. The higher the concentration of H_2_O_2_, the less the generation of these stress markers is favored.

For the concentrations of the two diastereoisomers of 10-F_4t_-NeuroP, none of the stress conditions applied appeared to supplant the control conditions.

Interestingly, despite the fact that the Mi124 species is known to have a high content of DHA, the concentrations of DHA-derived NeuroPs are not the most abundant nonenzymatic oxylipins found in this species.

#### 2.1.2. Profiles of Enzymatic Oxygenated Metabolites

##### Derivatives of LA (C18:2n−6)

The oxidized derivatives of omega-6 are mainly composed of derivatives of LA, specifically the following two hydroxyoctadecadienoic acids (HODEs): 13-HODE and 9-HODE. The analysis of these initial results seems to indicate a slight improvement in the production of metabolites under the application of H_2_O_2_ stress at the highest concentration and, to a lesser extent, at a lower concentration. Furthermore, the results appear to highlight the deleterious effect of other stressors on the production of the two derivatives in the HODE series.

##### Derivatives of ARA (C20:4n−6)

The derivatives of ARA, such as HETEs and EETs, were found at concentrations up 20 to 40 times less than the derivatives of LA.

The ARA derivatives exhibited a similar trend as the LA derivatives, with a high concentration observed at 4 mM of H_2_O_2_, which seems to be twice as high as the control. This was particularly the case for the 15-HETE. Similarly to the derivatives of LA, those of ARA seemed to decrease under nutritional and saline stress conditions.

##### Derivatives from ALA, EPA, and DHA

Five oxygenated metabolites resulting from the enzymatic oxidation of omega-3 PUFAs were identified. Among these metabolites, one derivative originates from ALA (e.g., 9(*R*),16(*RS*)-linotrin) and one from EPA (e.g., 18-HEPE), while three other metabolites (14- and 17-HDoHE and Pdx) are derived from DHA.

Although none of the results were significantly different, the following trends emerged:

(i) The addition of H_2_O_2_ to the culture medium seemed to induce a slight increase in the concentration of the 9(*R*),16(*RS*)-linotrin while EtOAc or NaCl would have the opposite effect;
(ii) The addition of H_2_O_2_ (4 mM) would also slightly increase the concentration of 18-HEPE, a derivative of EPA;
(iii) None of them appear to be produced in greater quantities when abiotic stresses are applied, since the highest concentrations for each of the metabolites appeared to be obtained with the control culture medium.

### 2.2. Profiles of Enzymatic and Nonenzymatic Oxygenated Metabolites in Stressed Mi136

**Figure 3:**
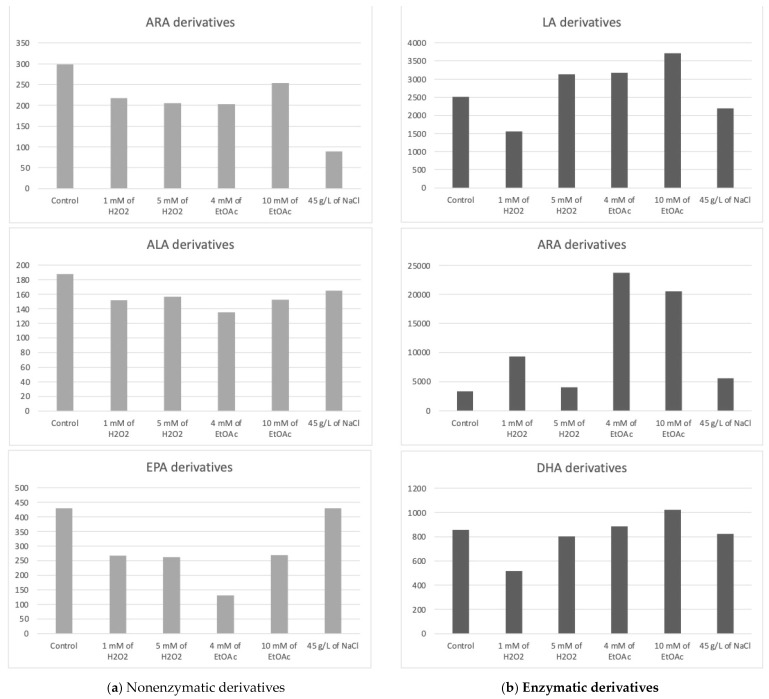
Sums of the concentrations, expressed in pg/mg DWB, of oxygenated metabolites classified by PUFAs in Mi136 depending on the growing conditions; control condition versus stress-inducing conditions (H_2_O_2_ at 1 mM or 5 mM; EtOAc at 4 or 10 mM; and NaCl at 45 g/L).

The chlorophyte “Mi136” distinguished itself with its abundance of EPA [6,48,49]. The concentration of PUFA-derived oxygenated metabolites varied only very slightly for all culture conditions. We counted 18 nonenzymatic oxylipins and 17 enzymatic oxylipins [6]. In Table 2, all concentrations of the various metabolites measured according to the growth conditions of the microalgae are listed. Figure 3, on the other hand, graphically represents these same values, aggregated by the PUFA series from which the compounds are derive.

#### 2.2.1. Profiles of Nonenzymatic Oxygenated Metabolites

##### Derivatives from ARA

Two ARA derivatives, 15-A_2_-IsoP and 5(*RS*)-5-F_2c_-IsoP, the resulted from an equimolar mixture of two epimers of the 5-F_2c_-IsoP, were detected for each of the culture conditions.

The trend observed from the replicates is as follows: the control culture conditions appeared to be the most favorable for the synthesis of nonenzymatic oxylipins from ARA.

Interestingly, under saline stress, the concentration of these oxylipins appeared to decrease, highlighting the unfavorable effect of this condition.

##### Derivatives from ALA

The nonenzymatic isoprostanoid and isofuranoid derivatives of ALA were detected and quantified for each condition, except for 9-F_1t_-PhytoP, which was at the limit of detection when growing in the presence of EtOAc.

It is interesting to note that among the PhytoPs (Table 2), 16-*epi*-16-F_1t_-PhytoP and 16-F_1t_-PhytoP were detected in higher concentrations compared to the 9-L_1t_-PhytoP, 9-F_1t_-PhytoP, and 16-B_1t_-PhytoP, with a ratio of at least 10:1.

Additionally, the concentrations of these type-F metabolites from the 16-series seemed to slightly increase in the presence of 5 mM of H_2_O_2_, while the 9-L_1t_-PhytoP, 9-F_1t_-PhytoP, and 16-B_1t_-PhytoP concentrations seemed to show the maximum increase under 1 mM H_2_O_2_ or NaCl stress.

As for PhytoFs, the measured PhytoFs exhibited similar profiles across all culture conditions, with the exception of *ent*-16A-13-epi-ST-Δ^14^-10-PhytoF, whose content appeared to decrease under abiotic stress conditions.

##### Derivatives from EPA

We quantified five nonenzymatic derivatives that formed from the oxidation of EPA. The total concentration of the F_3t_-IsoPs seemed to be the highest under both the control and saline stress conditions (Figure 3). The application of other abiotic stresses appeared to negatively impact the production of these nonenzymatic oxylipins.

Overall, the control condition often represented the most advantageous setting for producing nonenzymatic oxylipins in Mi136, and oxidative, nutritional, and saline stresses might not significantly increase the concentrations of these bioactive derivatives in this microalgal species.

#### 2.2.2. Profiles of the Enzymatic Oxygenated Metabolites

##### Derivatives from LA

Two enzymatic oxygenated metabolites of LA, 9-HODE and 13-HODE, were found in all culture conditions.

It appears that a slight increase in these derivatives’ production may be achieved through stress induction with EtOAc (10 mM) compared to the production levels observed under control conditions. However, this observation represents only a trend, as the differences were not statistically significant.

##### Derivatives from ARA

Ten enzymatic derivatives of ARA were quantified, among which were the monohydroxylated oxylipins (HETEs), epoxides oxylipins (EETs), and two more complexes and end-structures, the PGs.

Depending on the culture conditions, three of the HETEs exhibited significant variations in concentration. Unfortunately, these significant differences cannot be fully exploited, as they relate to comparisons between two abiotic stress conditions and not with the control condition. The study showed no significant difference for EET derivatives no matter the conditions applied. It would appear that the condition for which the cumulative levels of HETEs and EETs were the highest in these preliminary experiments was the control condition.

The PGE_2_ appeared the highest in the presence of EtOAc, whereas the 15-*d*-PGJ_2_ did not follow the same tendency, reaching its maximum production in the presence of 1 mM of H_2_O_2_. Because these two metabolites are present in greater quantities than the HETEs and EETs, they have a significant impact on the summary graph (Figure 3).

##### Derivatives from EPA

Two derivatives of EPA were detected, PGE_3_ and 18-HEPE. These metabolites appear to be slightly sensitive to variations in culture conditions for Mi136. They seemed to be in low concentrations under the control conditions. We point out, once again, that these are trends only, as the differences are not statistically significant. The PGE_3_ concentrations appeared to increase with the application of EtOAc, followed by 1 mM of H_2_O_2_. The two lower doses of EtOAc and H_2_O_2_ seemed to produce 18-HEPE at random, which accounts for the variability seen in the triplicate (large standard deviation).

##### Derivatives from DHA

The following two monohydroxylated metabolites of DHA, also called hydroxydocosahexaenoic acid (HDoHE), were found in the Mi136 species: 14-HDoHE and 17-HDoHE.

Interestingly, the 17-HDoHE oxylipin, which is the main precursor to PDx and NPD_1_ pro-resolvin metabolites, was found in higher quantities than its 14-series counterpart. It appears that the stresses induced in this study might slightly influence the production of the 14-HDoHE metabolite, potentially increasing its levels under H_2_O_2_ conditions, while other stresses seem to decrease its formation. As for the 17-HDoHE monohydroxylated oxylipin, the tested conditions appear to generally result in reduced production compared to the control conditions.

### 2.3. Profiles of Enzymatic and Nonenzymatic Oxygenated Metabolites in Stressed Mi168

**Figure 4:**
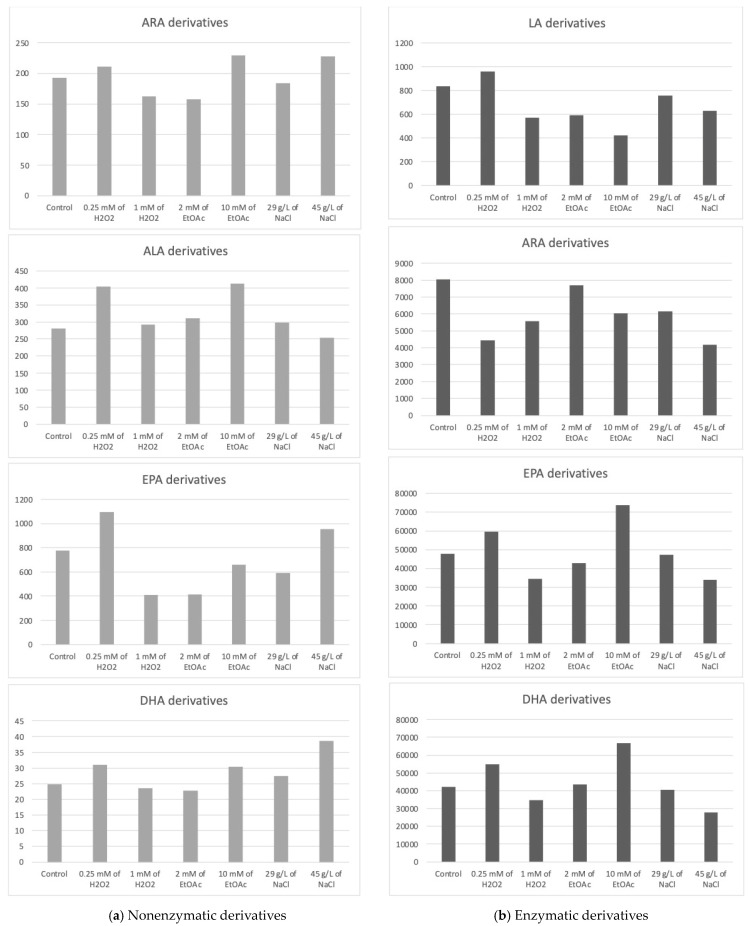
Sums of the concentrations, expressed in pg/mg DWB, of oxygenated metabolites classified by PUFAs in Mi168 depending on the growing conditions; control condition versus stress-inducing conditions (H_2_O_2_ at 0.25 mM or 1 mM; EtOAc at 2 or 10 mM; and NaCl at 29 g/L or 45 g/L).

The Haptophyte species “Mi168” distinguished itself with a substantial amount of EPA and DHA [6,43]. This species has 21 enzymatic and 27 nonenzymatic oxylipins, according to the analysis of its oxylipin profile [6]. Depending on the cultivation conditions, different metabolite levels seems to emerge. In Table 3, the concentrations of various metabolites according to the growth conditions of Mi168 are presented. Figure 4 shows the sums of these values, grouped by the precursor PUFAs, and categorized based on the type of oxidation—nonenzymatic on the one hand and enzymatic on the other.

#### 2.3.1. Nonenzymatic Oxygenated Metabolite Profiles

##### Derivatives from ARA

Once again, the nonenzymatic derivatives from ARA were identified and quantified for each culture condition, of which there were five.

The average total concentration of ARA derivatives appeared to be slightly higher with the addition of 10 mM of EtOAc or 45 g/L of NaCl without observing a statistically significant difference from the control.

##### Derivatives from DPA

The 4(*RS*)-4-F_3t_-NeuroP was detected and measured for each culture condition. This compound was not detected or quantified in the other two species studied.

The high production of 4(*RS*)-4-F_3t_-NeuroP seemed to be induced by the addition of 45 g/L NaCl in the culture medium.

##### Derivatives from ALA

The nonenzymatic ALA derivatives, PhytoPs and PhytoFs, were found in quantities of four and three, respectively. In this species, the ALA nonenzymatic oxylipin profiles, again, did not vary in a statistically significant way. We can only report on the trends.

16-F_1t_-PhytoP and 16-*epi*-16-F_1t_-PhytoP were found in higher concentrations than 9-L_1t_-PhytoP and 16-B_1t_-PhytoP. The total concentration of ALA derivatives seemed the highest with 0.25 mM of H_2_O_2_ or 10 mM of EtOAc.

##### Derivatives from EPA

Six nonenzymatic oxylipins from EPA were detected in the Mi168 species, which is one more metabolite than in the Mi136 species and three additional metabolites compared to Mi124.

The average concentration of the EPA derivative, F_3t_-IsoP, seemed to vary somewhat according to the stress conditions. Here again, the observable trend was a slight increase in the production of the derivative when stress was induced by a low concentration of H_2_O_2_.

##### Derivatives from DHA

Five metabolites derived from DHA were quantified, with 4(*RS*)-4-F_4t_-NeuroP being predominant under saline stress (45 g/L).

The total concentration of DHA derivatives also seemed higher under saline stress induced by 45 g/L of NaCl in the culture medium, followed by the EtOAc (10 mM) and H_2_O_2_ (0.25 mM) conditions.

#### 2.3.2. Enzymatic Oxygenated Metabolites Profiles

##### Derivatives from LA

Two HODEs, 9-HODE and 13-HODE, were identified as LA derivatives under all seven culture conditions.

The total concentration of HODEs (9- and 13-HODEs) seemed to increase very slightly when the culture was subjected to stress mediated by 0.25 Mm of H_2_O_2_, whereas it seemed to decrease when a higher concentration of H_2_O_2_ was applied or when a small concentration of ethyl acetate was added to the medium.

##### Derivatives from ARA

There were 12 oxidized derivatives of ARA, with five HETE, three EET, one TxB, and three PGs.

Once again, we cannot clearly distinguish the benefit of applying stress versus not applying it with the goal of increasing the production of oxidized metabolites. In the absence of a significant difference, we can only observe slight decreases compared to the control. Therefore, abiotic stresses do not seem to present any benefit in this case.

##### Derivatives from EPA

As for the other species, two EPA metabolites were detected.

Looking at the PGE3 profile, it would appear that two conditions are capable of increasing the synthesis of this derivative, namely, the highest concentration of EtOAc and, to a lesser extent, the lowest concentration of H_2_O_2_. Analysis of the data for 18-HEPE seems to lead to the same conclusion.

##### Derivatives from DHA

Two monohydroxylated oxylipins (HDoHEs), as well as a resolvin (RvD_5_) and a protectin (PDx), were found in the Mi168 species.

The enzymatic derivatives of DHA seemed to show the same trend as the EPA derivatives, with an increase in their production in the presence of 0.25 mM H_2_O_2_ or 10 mM of EtOAc.

In the absence of statistically significant differences, we cannot be more definitive. It would be interesting to continue this study by increasing the number of trials, for example.

## 3. Discussion

Marine microalgae are posited to serve as a reservoir of bioactive lipid mediators, suggesting a potentially significant role in preventive health measures, notably in mitigating inflammation [6]. Research in microalgal biorefineries has shed light on how culture parameters influence the production of polyunsaturated fatty acids (PUFAs) [50,51,52,53,54], which may also be associated with oxidative stress induced by the accumulation of reactive oxygen species (ROS). For instance, salinity stress and H_2_O_2_ have been proven to induce oxidative stress and bring about substantial alterations in PUFA production in *Dunaliella salina* [52]. However, there is limited understanding regarding the effects of these abiotic parameters on derivatives of oxidized PUFAs. Our previous work [37] focused on *T. isochrysis lutea*, *Phaeodactylum tricornutum*, and *Rhodomonas salina* and showed that the nonenzymatic oxylipins production could be increased by inducing direct oxidative stress, whether through the addition of copper or H_2_O_2_.

The current investigation focuses on exploring various cultural conditions that may influence the production of oxylipins, both enzymatic and nonenzymatic, in three microalgae species (Mi124, Mi136, and Mi168). These microalgae species were selected, according to our previous work [6], based on their richness in various oxidized metabolites, especially DHA and EPA derivatives. Microalgae were cultivated by modulating their culture medium (with different concentrations of H_2_O_2_, EtOAc, or NaCl) to orient and promote the production of molecules of interest. The three species responded differently to the various stress conditions applied. Therefore, the selected doses are specific to each species, aiming to maximize cell viability while inducing a certain impact on the culture. Although microalgae culture conditions may have altered the synthesis of the oxidized metabolites somewhat, this was done in a species-dependent manner. Significant variability in the biological replicates was observed. Statistically significant differences are rare, primarily due to the high variability in the observable production across the three species. Consequently, we identified trends rather than clear-cut differences. This variability cannot be solely attributed to the low number of replicates. The only factor we can propose is the unequal access to the light source during the stress application. It would have been valuable to investigate this aspect further through additional experiments.

Although we are aware of the lack of reproducibility in the results obtained, which makes it impossible to draw firm conclusions, we focus on highlighting the observed trends. These trends have, in fact, guided our decision to further investigate these species on a larger scale.

The most favorable condition for Mi124 to potentially achieve a significant concentration of both nonenzymatic and enzymatic oxylipins appears to involve inducing stress by adding H_2_O_2_ to the medium. These observations align with some previous work that reported an enhancement in the oxylipin production in culture conditions using H_2_O_2_ [32,34,37]. Accordingly, H_2_O_2_ might be considered a promising option for promoting oxylipin production while taking advantage of its antimicrobial properties [55]. Our findings revealed that a low dose (1 mM of H_2_O_2_) may lead to an increase in the generation of nonenzymatic oxylipins, while a high dose (4 mM) resulted in a concentration of enzymatic oxylipins comparable to control conditions. The synthesis of oxidized enzymatic derivatives from omega-6 appears to be particularly favored when 4 mM of H_2_O_2_ is added to the culture medium of Mi124. Generally, the addition of EtOAc and increasing the concentration of NaCl in the culture medium seem to correspond with a decrease in the production of enzymatic oxylipins by this species. Overall, the addition of 1 mM of H_2_O_2_ seems to increase the concentration of ARA and DPA derivatives. Moreover, nonenzymatic derivatives of ALA and EPA are produced in greater quantity in the presence of a low concentration of H_2_O_2_ (1 mM) or EtOAc (0.25 mM) in the culture medium of Mi124.

For Mi136, we were unable to identify conditions that significantly boosted oxylipin levels beyond those observed in the control condition. Regarding enzymatic metabolites, the presence of 10 mM of EtOAc appeared to lead to a targeted increase in DHA derivatives by 30% compared to the control. However, the control condition still seems to be the most favorable for obtaining a high content of nonenzymatic oxylipins. Nevertheless, with 45 g/L of NaCl in the culture medium, there was an observed increase in the selective production of diastereoisomers of 5-F_3t_-IsoP by almost 30% compared to the control condition.

Mi168 is the microalgae that produces the greatest diversity of oxylipins. Furthermore, during the experiments, this species demonstrated a notable increase in oxylipin production under the stress conditions compared to the control conditions. The “salt stress” condition at 45 g/L may be a potentially favorable condition for increasing nonenzymatic oxylipins, although it also seems to significantly decreases the synthesis of enzymatic oxylipins. Indeed, salt stress alters osmotic pressure, resulting in an ionic imbalance that induces oxylipins production and activates the PLA2 enzyme, promoting lipid liberation and then lipid peroxidation [42,56], which explains why the oxylipins increase. Stress induced by the addition of 0.25 mM H_2_O_2_ or 10 mM EtOAc has been associated with an overall increase in both nonenzymatic and enzymatic oxylipins. Indeed, under these conditions, there was a notable increase in HODEs, metabolites involved in signaling and regulating inflammatory processes [57], and 18-HEPE, which reduces the pro-inflammatory activity of myocardial fibroblasts [58] and resistance to some viral infections [59]. These conditions also seem to promote the production of PhytoPs and PhytoFs, which are involved in regulating immune function, combating certain types of cancers, and exerting vasoconstrictive effects [60].

Aside from the main goal of this paper, which is to determine the optimal conditions for producing high oxylipin quantities, we discovered that even when investigating a species rich in specific PUFAs, increased production of derivatives from other PUFAs was possible, with oxylipins resulting from PUFAs that were not defined as the majority and, therefore, not necessarily listed among the PUFAs of the species.

For instance, Mi124 is mainly rich in omega-3 PUFAs, especially DHA and ALA [6,46,47], and our findings reveal a high level of production in oxidized metabolites of enzymatic DHA followed by nonenzymatic ALA, indicating a logical correlation (Figure 4). However, we also demonstrated a 10-fold lower production of other PUFA metabolites than the first listed metabolites, including enzymatic ARA derivatives and nonenzymatic EPA derivatives, which were not present or reported in the Mi124 matrix. Additionally, enzymatic LA derivatives were detected in the oxylipins analysis. This finding raises questions concerning the prevalence of these PUFAs in the matrix structure, particularly omega-6 PUFAs.

Mi136 was reported to have an abundance of EPA in its matrix [6,48,49] and produced high levels of both enzymatic and nonenzymatic EPA. However, a high amount of nonenzymatic ALA derivatives, as well as enzymatic ARA and LA derivatives, were measured. Mi168 is recognized for being high in DHA and EPA [6,43] omega-3 PUFAs, and our findings demonstrated a high-level of enzymatic EPA and DHA derivatives generation followed by nonenzymatic EPA derivatives. Nevertheless, the amounts of enzymatic LA derivatives were high, comparable to the value of the latter metabolites. Also, nonenzymatic ALA metabolites were detected.

Overall, several metabolites were detected and quantified in larger quantities, even though they were derived from PUFAs that were not reported in the matrix species. This fact underscores, in our view, the difficulty of analyzing polyunsaturated fatty acids (PUFAs) in microalgal matrices and the importance of a well-considered sample preparation (especially extraction), which is essential for a complete PUFAs profile. Without the most exhaustive possible analysis of the PUFA contents in the starting matrix, any correlations between PUFAs and the oxylipins derived from them are impossible to identify.

## 4. Materials and Methods

In this study, we conducted a comparative analysis of oxylipins in three distinct marine microalgae species cultivated in 1 L volumes under various stress conditions. For each of the microalgae species, we differentiated between enzymatic and nonenzymatic oxylipins. The three species are characterized and compared in terms of their oxylipin profiles under stress conditions. The statistical analysis was performed using GraphPad Prism version 9.5. Significant differences were calculated using an ANOVA with the Tukey method to compare each condition.

### 4.1. Chemicals and Reagents

Commercially isoprostanoid standards (2,3-*dinor*-15-F_2t_-IsoP) and the internal standard (ISTD) d4-15-F_2t_-IsoP were obtained from Cayman Chemicals.

Other isoprostanoid standards and internal standards (d4-10-F_4t_-NeuroP, C19-16-F_1t_-PhytoP, and C21-15-F_2t_-IsoP) were synthesized following previous procedures [13], including phytoprostanoids (9-F_1t_-PhytoP, *ent*-16-*epi*-16-F_1t_-PhytoP, *ent*-16-F_1t_-PhytoP, and 9-L_1t_-PhytoP) and phytofuranes (*ent*-16A-13-*epi*-ST-∆^14^-9-PhytoF, *ent*-16B-13-*epi*-ST-∆^14^-9-PhytoF, *ent*-16A-9-*epi*-ST-∆^14^-10-PhytoF, *ent*-16B-9-*epi*-ST-∆^14^-10-PhytoF, *ent*-9A-12-*epi*-ST-∆^10^-13-PhytoF, and *ent*-9B-12-*epi*-ST-∆^10^-13-PhytoF) obtained from the oxidation of α-linolenic acid (ALA), C18:3n−3; isoprostanoids derived from arachidonic acid (ARA), C20:4n−6 (15-A_2t_-IsoP, 5-F_2c_-IsoP, 5-*epi*-5-F_2t_-IsoP, and 5-F_2t_-IsoP); isoprostanoids derived from ecosapentaenoic acid (EPA), C20:5n−3 (5(*S*)-5-F_3t_-IsoP, 8(*R*)-8-F_3t_-IsoP, 8(*S*)-8-F_3t_-IsoP, 18(*R*)-18-F_3t_-IsoP, and 18(*S*)-18-F_3t_-IsoP); and neuroprostanoids (4(*RS*)-4-F_4t_-NeuroP, 10(*R*)-10-F_4t_-NeuroP, 10(*S*)-10-F_4t_-NeuroP, 13(A)-13-F_4t_-NeuroP, 13(B)-13-F_4t_-NeuroP, 14(*R*)-14-F_4t_-NeuroP, 14(*S*)-14-F_4t_-NeuroP, and 20(*R*)-20-F_4t_-NeuroP) from docosahexaenoic acid (DHA), C22:6n−3; and, finally, 4(*RS*)-4-F_3t_-NeuroP, obtained from the oxidation of docosapentaenoic acid (DPA), C22:5n−3. “A” and “B” designate the R or S configuration, but these were not determined.

For the “enzymatic oxylipins”, 6-keto-prostaglandin F1 (6kPGF1), thromboxane B2 (TXB2), prostaglandin E2 (PGE2), prostaglandin E3 (PGE3), lipoxin A4 (LxA4), lipoxin B4 (LxB4), deuterated lipoxin A4 (LxA4-d5), resolvin D1 (RvD1), 7(S)-maresin (7-MaR1), leukotriene B4 (LTB4), leukotriene B5 (LTB5), deuterated leukotriene B4 (LTB4-d4), 10(S),17(S)-protectin (PDx), 18-hydroxyeicosapentaenoic acid (18-HEPE), dihydroxy-eicosatetraenoic acid (5,6-DiHETE), 15-hydroxyeicosatetraenoic acid (15-HETE), and 12-HETE, 8-HETE, 5-HETE, 5-HETE-d8, 19-HETE, 20-HETE, 17-hydroxy-docosahexaenoic acid (17-HDoHE), 14-HDoHE, 14,15-epoxyeicosatrienoic acid (14,15-EET), and 11,12-EET, 8,9-EET, 5,6-EET, 5-oxoeicosatetraenoic acid (5-oxo-ETE), and zileuton were purchased from Cayman Chemicals (Ann Arbor, MI, USA).

Chloroform (CHCl_3_, HPLC grade), acetonitrile (ACN, LC-MS grade), methanol (MeOH, LC-MS grade), and water purified with a milli-Q system (H_2_O, LC-MS grade), were purchased from Fisher Scientific.

Butylated hydroxytoluene (BHT), absolute ethanol (EtOH, HPLC grade), hexane (HPLC grade), formic and acetic acids (HCOOH and AcOH, HPLC grade) were obtained from Sigma Aldrich. Ammonia (NH_3_, 28%) and ethyl acetate (EtOAc) (>99.8%) were bought from VWR. Hank’s Balanced Salt Solution (HBSS), phorbol myristate acetate (PMA), and zymosan A (ZYM) and A23187 were from Sigma–Aldrich (Saint Quentin Fallavier, France).

Solid-phase extraction cartridges (Oasis^®^ MAX Cartridge, 60 mg) were obtained from Waters.

### 4.2. Microalgal Species and Nonstressful Conditions

Three confidential species of marine microalgae were selected based on their rich profile/high concentration of oxylipins of interest, as demonstrated in our previous work [6]. These species include two *Haptophytes* and one *Chlorophyte*. The two Haptophytes are referred to as “Mi124” and “Mi168” and have been shown to have a high level of oxygenated metabolites of DHA. The *Chlorophyte*, on the other hand, demonstrated a greater profile of oxylipins derived from EPA and is also referred to as “Mi136”.

Under nonstressful conditions, these marine microalgae were grown in photobioreactors at a controlled temperature of 24 °C, using a medium based on f/2 medium [61] supplemented with artificial sea water at a concentration of 15 g/L. The pH of the medium was maintained between 7.5 and 8, while the incoming air was enriched with 3% CO_2_ at an average flowrate of 3 L/h. The microalgae were exposed to a constant light intensity of 50 µmol·m^−2^·s^−1^ provided by daylight LEDs, with continuous 24/0 lighting throughout the growth period.

### 4.3. Viability Cell Tests

To assess the viability of the cells, it was crucial to evaluate the various stress doses before implementing a stress condition. Viability tests were performed using sterile culture multiwell plates, 24 wells, Grynia (VWR, Paris, France), in order to make a first selection of stress doses applicable to microalgae. Different quantities of H_2_O_2_ or EtOAc were added with the concentrations in the range of 0 mM to 10 mM·L^−1^. Concentrations of 0, 15, 25, and 45 g/L of NaCl were tested for their effects on osmotic stress. These concentrations were selected based on their feasibility for implementation on an industrial scale.

The optical density and microscopic monitoring were conducted over a period of five days to evaluate the impact of stress on cell viability. Two doses (low and high) were chosen for the H_2_O_2_ and EtOAc conditions per species based on the highest observed viability. Two doses of NaCl were applied to the Mi168, while only the highest dose was used for the Mi124 and Mi136.

For Mi124, the tolerated doses were determined as 1 mM and 4 mM for H_2_O_2_, 0.25 mM and 1 mM for EtOAc, and 45 g/L for NaCl. The Mi136 exhibited enhanced cell viability with 1 mM and 5 mM additions of H_2_O_2_, 4 mM and 10 mM additions of EtOAc, and 45 g/L of NaCl. Lastly, the selected doses for the Mi168 were determined as 0.25 mM and 1mM for H_2_O_2_, 2 mM and 10 mM for EtOAc, and 29 g/L and 45 g/L for NaCl.

Subsequently, fresh biomass from stressed cultures under the selected conditions was analyzed for oxylipin content.

### 4.4. Stress Induction

**Figure 5:**
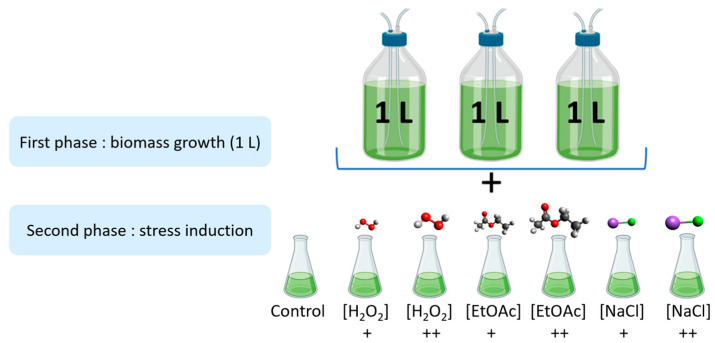
Experimental design of the stress inductions (+: Low dose; ++: high dose).

As shown in Figure 5, the stress induction was carried out in two parts. Firstly, microalgae were cultivated in biological triplicates under nonstressful conditions in 1 L photobioreactors at Microphyt’s manufacturing facility in Baillargues, France. During the mid-growth phase, the culture was exposed to stress conditions by adding two different levels of H_2_O_2_, EtOAc, and NaCl for 48 h before harvesting.

The cells were harvested by centrifugation at 6000 rpm (Rotixa 500 RS, Hettich, Kirchlengern, Germany) and subsequently frozen at −80 °C until analysis.

### 4.5. Nonenzymatic Oxylipins Extraction and Analysis

Nonenzymatic oxylipins were extracted and analyzed using the same protocol as previously published in prior work [6]. Samples (100 mg of fresh biomass) with 25 µL of BHT were ground in lysing matrix tubes (lysing matrix D, MP Biochemicals, Illkirch, France) with 1 mL of MeOH and 4 µL of ISTD mix (1 ng/μL) using a FastPrep-24 (MP Biochemicals) for 30 s at a speed of 6.5 m·s^−1^. The suspensions were transferred to clean centrifuge tubes (15 mL) with 1 mL of MeOH and 1.5 mL of phosphate buffer (50 mM, pH 2.1) saturated with NaCl. The tubes were then stirred for 30 min at room temperature. Subsequently, the mixture was centrifuged at 5000 rpm for 5 min at room temperature. The supernatant was recovered, and 4 mL of chloroform was added and stirred for 30 s before being centrifuged at 2000 rpm for 5 min at 4 °C. The organic phase was collected in Pyrex tubes and concentrated under N_2_ for an average of 1 h at 40 °C in a dry bath. Hydrolysis was performed with 950 µL of 1 M KOH for 30 min at 40 °C. After incubation, 1 mL of 40 mM formic acid was added before starting the solid-phase extraction (SPE) on a Biotage^®^ Extrahera^TM^, an automatic sample preparation system. Samples were then loaded onto pre-conditioned Oasis MAX cartridges and successively washed with 1.5 mL of NH_3_ 2% (*v*/*v*), 1.5 mL of MeOH/20 mM formic acid (30:70; *v*/*v*), 1.5 mL of hexane, and 1.5 mL of hexane/ethyl acetate (70:30; *v*/*v*). The retained oxylipins were eluted by adding 2 mL of hexane/EtOH/acetic acid (70:29.4:0.6; *v*/*v*/*v*). The samples were concentrated under a nitrogen flow for an average of 1 h at 40 °C in a dry bath and reconstituted in 100 μL of mobile-phase solvents (H_2_O/ACN; 83:17; *v*/*v*) for injection.

All of the LC-MS/MS analyses were conducted using an Eksigent micro high-performance liquid chromatography (HPLC) 200 Plus from Sciex Applied Biosystems, Framingham, MA, USA. The system was equipped with a HALO C18 analytical column (100 × 0.5 mm, 2.7 μm; Eksigent Technologies, CA, USA), which was maintained at a temperature of 40 °C. The mobile phase used in the LC-MS/MS analysis consisted of a binary gradient of two solvents—solvent A was composed of H_2_O with 0.1% (*v*/*v*) HCOOH, while solvent B was composed of ACN with 0.1% (*v*/*v*) HCOOH. The flow rate used was 0.03 mL·min^−1^, and the injection volume was 5 µL. The elution gradient was as follows: 17% B at 0 min, 17% B at 2.6 min, 21% B at 2.85 min, 25% B at 7.3 min, 28.5% B at 8.8 min, 33.3% B at 11 min, 40% B at 15 min, and 95% B at 16.5 min for 1.5 min. An AB SCIEX QTRAP 5500 (Sciex Applied Biosystems, Vaughan, ON, Canada) was used to perform mass spectrometry analyses. Electrospray (ESI) was employed as the ionization source, operating in the negative mode. The source voltage was maintained at −4.5 kV, and N_2_ was utilized as the curtain gas. Detection of the fragmentation ion products from each deprotonated molecule [M − H]^−^ was carried out in the multiple reaction monitoring mode (MRM) (Table S1, supplementary data), with individually optimized MS parameters for each compound. The mass spectrometer was operated using Analyst^®^ software (Sciex Applied Biosystems) for the LC-MS/MS data acquisition. MultiQuant 3.0 software (Sciex Applied Biosystems) was used for peak integration and quantification of analytes.

### 4.6. Enzymatic Oxylipins Extraction and Analysis

Deuterium-labeled eicosanoids (LxA4-d5, LTB4-d4, and 5-HETEd8) were mixed at a concentration of 400 ng/mL in MeOH and used in the internal standard (IS) solution. Stock solutions of PUFA metabolites were prepared in MeOH at a concentration of 2000 ng/mL for each compound and stored in a glass vial at −80 °C. Ten successive dilutions by half were prepared in MeOH. These 10 solutions were mixed with the IS solution at a ratio of 1:1 to prepare the following standard solutions: 1.95, 3.9, 7.8, 15.6, 31.25, 62.5, 125, 250, and 500 ng/mL containing 200 ng/mL of IS.

A protocol similar to the one described by P. Le Faouder [62] and described in detail in our prior research work [6] was used to quantify the enzymatic oxylipins at the MetaToul lipidomic facility.

Briefly, fresh biomass (25 mg) and 500 μL of HBSS were homogenized with a FastPrep^®^ Instrument (MP Biomedicals, LLC, Santa Ana, CA, USA). The resulting homogenate was mixed with 260 μL of cold MeOH and 40 μL of IS solution (50 ng/mL) and then centrifuged at 5000 rpm for 15 min at 4 °C. The supernatants were collected, diluted to 2 mL with H_2_O, and subjected to solid-phase extraction using an HRX-50 mg 96-well clusters (Macherey Nagel, Hoerd, France). The sample was loaded onto the plate after conditioning with 1 mL of MeOH and 1 mL of H_2_O/MeOH (90:10, *v*/*v*). The plate was washed with 1 mL of H_2_O/MeOH (90:10, *v*/*v*), and the lipid mediators were eluted with 1 mL of MeOH. The samples were then evaporated in a dry bath at 40 °C under a nitrogen flow. The resulting dried extracts were dissolved in 10 μL of MeOH, and 5 μL of the resulting extract was injected and analyzed using LC-MS/MS. Analyst^®^ software (Sciex Applied Biosystems) was used to acquire the LC-MS/MS data, while MultiQuant 3.0 software (Sciex Applied Biosystems) was used for peak integration and quantification of the analytes.

## 5. Conclusions

Microalgae are regarded as a rich source of PUFAs (omega-3 and omega-6), which produce oxylipins through lipid peroxidation. Understanding the mechanisms underlying oxylipin production in microalgae, as well as the factors that influence their accumulation, can be valuable for devising strategies to optimize their production for diverse applications.

Microalgae exhibit tremendous cellular plasticity, with large alterations in their biochemical composition in response to environmental restrictions. Oxylipins, which are indicators of oxidative stress, appear to exhibit different generative patterns based on the culture conditions for the three studied microalgae species. However, this observation is species-specific, as each microalgae species possesses a distinct stress tolerance, resulting in varying qualitative and quantitative oxylipin profiles.

In the current study, the most favorable conditions in the culture medium for increasing oxylipin production, both enzymatic and nonenzymatic, were the following: (i) adding H_2_O_2_ to the medium for Mi124; (ii) keeping the control condition medium for Mi136; (iii) for Mi168 the answer is less clear-cut, since the best conditions fluctuate according to the series of oxylipins.

In the future, efforts will be directed at cultivating microalgae on an industrial scale using stress conditions explored on a 1 L scale. The goal is to develop bioactive ingredients derived from microalgae enriched in oxylipins.

## Figures and Tables

**Table 1:** Quantification of nonenzymatic and enzymatic oxygenated metabolites in Mi124.

Mi124																		
Oxylipins	Control			1 mM of H_2_O_2_			4 mM of H_2_O_2_			0.5 mM of EtOAc			1 mΜ of EtOAc			45 g/L of NaCl		
	Concentration (pg/mg)	±	SD	Concentration (pg/mg)	±	SD	Concentration (pg/mg)	±	SD	Concentration (pg/mg)	±	SD	Concentration (pg/mg)	±	SD	Concentration (pg/mg)	±	SD
9-HODE	4.58 × 10^3^	±	3.12 × 10^2^	9.14 × 10^3^	±	5.95 × 10^2^	9.33 × 10^3^	±	1.87 × 10^3^	3.87 × 10^3^	±	6.53 × 10^2^	3.15 × 10^3^	±	8.08 × 10^2^	3.09 × 10^3^	±	3.53 × 10^2^
13-HODΕ	1.38 × 10^4^	±	3.00 × 10^3^	1.40 × 10^4^	±	1.76 × 10^3^	1.88 × 10^4^	±	6.97 × 10^3^	1.11 × 10^4^	±	5.13 × 10^3^	9.71 × 10^3^	±	5.71 × 10^3^	7.50 × 10^3^	±	2.08 × 10^3^
5oxoETE	3.12 × 10	±	9.24	3.04 × 10	±	2.60 × 10	9.28 × 10	±	2.05 × 10	1.97 × 10	±	1.47 × 10	1.78 × 10	±	1.50 × 10	2.07 × 10	±	8.41
5-HΕΤΕ	3.95 × 10	±	2.02	5.55 × 10	±	2.54	1.52 × 10^2^	±	4.34 × 10	3.18 × 10	±	1.27 × 10	2.11 × 10	±	1.10 × 10	2.40 × 10	±	8.37
8-HΕΤΕ	2.19 × 10	±	8.33	2.72 × 10	±	2.10	6.46 × 10	±	1.62 × 10	1.79 × 10	±	9.25	1.43 × 10	±	1.10 × 10	1.22 × 10	±	2.55
12-HΕΤΕ	3.18 × 10	±	1.19 × 10	4.83 × 10	±	1.14 × 10	1.21 × 10^2^	±	2.24 × 10	3.13 × 10	±	2.35 × 10	1.88 × 10	±	1.55 × 10	1.12 × 10	±	8.60
15-HΕΤΕ	4.49 × 10^2^	±	1.23 × 10^2^	3.57 × 10^2^	±	1.01 × 10^2^	7.76 × 10^2^	±	3.42 × 10^2^	3.31 × 10^2^	±	2.03 × 10^2^	2.79 × 10^2^	±	1.96 × 10^2^	2.10 × 10^2^	±	8.19 × 10
14.15-ΕΕΤ	2.51 × 10	±	1.79 × 10	1.71 × 10	±	1.23 × 10	3.66 × 10	±	1.55 × 10	2.73 × 10	±	2.10 × 10	1.57 × 10	±	2.12 × 10	4.85	±	6.85
9(*R*).16(*R*.*S*)-Linotrines	4.30 × 10^2^	±	4.96 × 10	4.04 × 10^2^	±	9.40 × 10	4.24 × 10^2^	±	1.42 × 10^2^	2.89 × 10^2^	±	2.19 × 10	1.91 × 10^2^	±	6.39 × 10	2.7 × 10^2^	±	6.59 × 10
18-HEPE	8.55 × 10^2^	±	2.34 × 10^2^	7.53 × 10^2^	±	8.60	1.01 × 10^3^	±	2.36 × 10^2^	6.81 × 10^2^	±	2.53 × 10^2^	5.87 × 10^2^	±	4.02 × 10^2^	5.43 × 10^2^	±	2.08 × 10^2^
ΡDx	1.04 × 10^2^	±	5.17 × 10	3.83 × 10	±	1.03 × 10	4.85 × 10	±	2.48 × 10	8.87 × 10	±	5.57 × 10	8.42 × 10	±	9.81 × 10	3.15 × 10	±	1.94 × 10
14-HDoHE	1.07 × 10^4^	±	1.23 × 10^3^	1.15 × 10^4^	±	1.39 × 10^3^	1.33 × 10^4^	±	4.03 × 10^3^	8.65 × 10^3^	±	3.06 × 10^3^	6.27 × 10^3^	±	3.08 × 10^3^	6.20 × 10^3^	±	2.22 × 10^3^
17-HDoHE	1.22 × 10^5^	±	3.81 × 10^4^	9.10 × 10^4^	±	2.11 × 10^4^	9.06 × 10^4^	±	3.05 × 10^4^	9.31 × 10^4^	±	5.57 × 10^4^	8.07 × 10^4^	±	5.98 × 10^4^	6.28 × 10^4^	±	3.68 × 10^4^
9-L_1t_-PhytoP	65.31	±	11.53	90.89	±	7.24	89.25	±	1.89	50.80	±	6.89	66.48	±	12.64	59.95	±	8.57
9-F_1t_-PhytoP	32.88	±	2.31	64.26	±	17.70	54.69	±	4.54	28.53	±	5.43	30.30	±	1.57	49.25	±	12.30
16-B_1t_-PhytoP	64.90	±	9.96	94.93	±	8.60	92.14	±	2.15	52.04	±	6.88	66.24	±	11.28	70.40	±	11.31
16-*epi*-16-F_1t_-PhytoP	136.68	±	35.56	154.10	±	29.85	166.69	±	43.22	162.60	±	12.12	158.19	±	68.11	97.43	±	17.70
16-F_1t_-PhytoP	191.01	±	51.00	202.69	±	32.71	234.50	±	66.10	232.24	±	15.52	228.76	±	102.82	132.98	±	23.32
ent-9A-12-*epi*-ST-Δ^10^-13-PhytoF	44.25	±	13.08	62.05	±	3.04	55.00	±	4.73	33.29	±	7.76	54.11	±	14.81	40.92	±	4.58
ent-9B-12-*epi*-ST-Δ^10^-13-PhytoF	14.75	±	4.45	20.95	±	2.77	16.44	±	1.54	11.42	±	2.67	16.54	±	4.35	19.95	±	3.44
ent-16A-9-*epi*-ST-Δ^14^-10-PhytoF	95.51	±	31.37	105.70	±	11.86	94.80	±	18.53	66.76	±	14.37	122.56	±	47.00	49.93	±	6.80
ent-16B-9-*epi*-ST-Δ^14^-10-PhytoF	122.81	±	42.73	131.16	±	15.43	115.29	±	22.14	83.16	±	9.69	146.41	±	51.37	58.83	±	10.08
ent-16A-13-*epi*-ST-Δ^14^-9-PhytoF	15.75	±	1.21	21.30	±	2.10	20.83	±	0.90	13.70	±	3.21	14.77	±	2.42	16.24	±	1.21
ent-16B-13-*epi*-ST-Δ^14^-9-PhytoF	23.87	±	7.07	26.67	±	3.92	24.34	±	4.66	16.14	±	4.32	28.32	±	11.17	12.93	±	1.48
5(*R*)-5-F_3t_-lsoP	40.49	±	28.74	110.61	±	92.55	31.46	±	22.71	116.24	±	103.04	27.35	±	19.60	57.62	±	19.22
8(*R*)-8-F_3t_-lsoP	10.31	±	3.35	16.28	±	0.67	12.64	±	2.30	10.68	±	2.36	16.43	±	10.83	6.45	±	1.89
18(*R*)-8-F_3t_-IsoP	56.50	±	9.99	111.57	±	83.51	75.67	±	16.67	144.45	±	122.63	54.16	±	38.59	54.00	±	5.26
4(*RS*)-4-F_4t_-NeuroP	15.47	±	1.78	37.48	±	13.54	25.77	±	0.72	14.51	±	2.30	15.61	±	3.68	20.32	±	3.23
10(*R*)-10-F_4t_-NeuroP	27.06	±	18.53	12.61	±	3.63	15.96	±	3.46	18.54	±	4.06	20.37	±	9.16	14.45	±	0.90
10(*S*)-10-F_4t_-NeuroP	23.49	±	6.60	16.37	±	6.44	19.77	±	2.76	20.81	±	5.48	18.09	±	6.77	11.81	±	1.67
13A(*RS*)-13-F_4t_-NeuroP	16.55	±	3.76	28.61	±	6.14	24.18	±	2.19	16.01	±	4.49	20.80	±	2.72	19.71	±	2.50
13B(*RS*)-13-F_4t_-NeuroP	13.57	±	4.57	23.01	±	6.00	17.27	±	2.32	11.80	±	3.52	11.60	±	1.90	17.22	±	5.37

**Table 2:** Quantification of the nonenzymatic and enzymatic oxygenated metabolites in Mi136.

Mi136																		
Oxylipins	Control			1 mM of H_2_O_2_			5 mM of H_2_O_2_			4 mM of EtOAc			10 mΜ of EtOAc			45 g/L of NaCl		
	Concentration (pg/mg)	±	SD	Concentration (pg/mg)	±	SD	Concentration (pg/mg)	±	SD	Concentration (pg/mg)	±	SD	Concentration (pg/mg)	±	SD	Concentration (pg/mg)	±	SD
9-HODE	9.26 × 10^2^	±	1.54 × 10^2^	5.44 × 10^2^	±	1.24 × 10^2^	1.17 × 10^3^	±	1.46 × 10^2^	1.16 × 10^3^	±	2.54 × 10	1.31 × 10^3^	±	2.06 × 10^2^	8.48 × 10^2^	±	1.42 × 10^2^
13-HODΕ	1.59 × 10^3^	±	2.89 × 10^2^	1.01 × 10^3^	±	1.91 × 10^2^	1.96 × 10^3^	±	2.40 × 10^2^	2.02 × 10^3^	±	8.21 × 10	2.40 × 10^3^	±	2.86 × 10^2^	1.35 × 10^3^	±	2.66 × 10^2^
5-HΕΤΕ	2.49 × 10^2^	±	2.71 × 10	1.95 × 10^2^	±	7.63	3.11 × 10^2^	±	8.77	2.53 × 10^2^	±	8.86	2.20 × 10^2^	±	4.75 × 10	1.96 × 10^2^	±	4.23 × 10
8-HΕΤΕ	1.60 × 10^2^	±	1.23 × 10	1.17 × 10^2^	±	1.25 × 10	1.93 × 10^2^	±	1.11 × 10	1.79 × 10^2^	±	1.45 × 10	1.63 × 10^2^	±	3.34 × 10	1.31 × 10^2^	±	1.49 × 10
12-HΕΤΕ	2.82 × 10^2^	±	7.18	1.82 × 10^2^	±	2.46 × 10	2.84 × 10^2^	±	1.47 × 10	2.93 × 10^2^	±	2.03 × 10	2.77 × 10^2^	±	4.63 × 10	1.75 × 10^2^	±	2.93 × 10
15-HΕΤΕ	3.49 × 10^2^	±	3.70 × 10	2.85 × 10^2^	±	2.79 × 10	3.59 × 10^2^	±	1.42 × 10	3.83 × 10^2^	±	1.57	3.58 × 10^2^	±	4.86 × 10	1.98 × 10^2^	±	1.26 × 10
5oxoETE	1.19 × 10^2^	±	1.98 × 10	9.26 × 10	±	5.77	8.74 × 10	±	1.53 × 10	1.05 × 10^2^	±	1.07 × 10	1.07 × 10^2^	±	1.85 × 10	8.19 × 10	±	2.23 × 10
8.9-EET	1.35 × 10^2^	±	2.82 × 10	1.23 × 10^2^	±	2.32 × 10	1.38 × 10^2^	±	2.14 × 10	1.47 × 10^2^	±	1.85	1.42 × 10^2^	±	1.65 × 10	8.80 × 10	±	3.45 × 10
11.12-EET	1.50 × 10^2^	±	2.27 × 10	1.23 × 10^2^	±	2.48 × 10	1.30 × 10^2^	±	2.79 × 10	1.46 × 10^2^	±	1.74 × 10	1.13 × 10^2^	±	7.94	7.00 × 10	±	1.94 × 10
14.15-ΕΕΤ	1.43 × 10^2^	±	3.70 × 10	1.24 × 10^2^	±	3.57 × 10	1.10 × 10^2^	±	3.85 × 10	1.59 × 10^2^	±	9.89	1.16 × 10^2^	±	2.00 × 10	6.08 × 10	±	2.25 × 10
PGE_2_	1.80 × 10^2^	±	2.70 × 10^2^	3.64 × 10^2^	±	4.82 × 10^2^	9.01 × 10	±	8.28 × 10	1.04 × 10^3^	±	1.05 × 10^3^	6.87 × 10^2^	±	8.42 × 10^2^	3.42 × 10^2^	±	2.19 × 10^2^
PGE_3_	1.51 × 10^3^	±	1.54 × 10^3^	7.43 × 10^3^	±	4.02 × 10^3^	2.23 × 10^3^	±	2.07 × 10^3^	2.04 × 10^4^	±	8.99 × 10^3^	1.81 × 10^4^	±	1.64 × 10^4^	4.22 × 10^3^	±	5.71 × 10^3^
15-d-PGJ_2_	3.59 × 10	±	6.16 × 10	2.97 × 10^2^	±	1.69 × 10^3^	6.66 × 10	±	1.50 × 10	5.81 × 10^2^	±	1.08 × 10^3^	2.19 × 10^2^	±	3.44 × 10	4.21 × 10	±	1.12 × 10^2^
18-HEPE	4.73 × 10^4^	±	1.05 × 10^5^	2.36 × 10^5^	±	1.27 × 10^6^	6.69 × 10^4^	±	1.78 × 10^4^	6.82 × 10^5^	±	1.09 × 10^6^	2.62 × 10^5^	±	2.47 × 10^4^	1.26 × 10^5^	±	1.17 × 10^5^
14-HDoHE	3.73 × 10^2^	±	7.66 × 10	2.25 × 10^2^	±	5.08 × 10	3.61 × 10^2^	±	4.39 × 10	4.20 × 10^2^	±	2.67 × 10	4.66 × 10^2^	±	8.73 × 10	3.85 × 10^2^	±	5.08 × 10
17-HDoHE	4.83 × 10^2^	±	9.38 × 10	2.90 × 10^2^	±	8.94 × 10	4.40 × 10^2^	±	4.88 × 10	4.64 × 10^2^	±	5.74 × 10	5.56 × 10^2^	±	1.13 × 10^2^	4.38 × 10^2^	±	7.32 × 10
5(*RS*)-5-F_2t_-lsoP	167.17	±	89.06	115.41	±	40.19	109.59	±	10.60	97.28	±	20.92	116.45	±	49.26	55.15	±	8.06
15-A_2_-IsoP	130.51	±	46.56	101.89	±	21.26	94.74	±	7.68	105.64	±	19.85	138.11	±	55.59	34.07	±	7.63
9-L_1t_-PhytoP	6.55	±	4.64	3.50	±	0.94	3.05	±	1.10	2.98	±	0.73	4.64	±	1.12	9.00	±	10.73
9-F_1t_-PhytoP	8.97	±	12.69	3.19	±	4.51	2.23	±	3.15	0.00	±	0.00	0.00	±	0.00	4.69	±	6.63
16-B_1t_-PhytoP	8.43	±	7.14	4.35	±	1.43	3.57	±	1.35	3.54	±	0.66	4.70	±	1.23	11.24	±	13.06
16-*epi*-16-F_1t_-PhytoP	57.05	±	3.84	49.25	±	8.16	56.88	±	9.46	45.42	±	11.67	50.85	±	3.25	52.50	±	6.67
16-F_1t_-PhytoP	68.99	±	5.64	62.36	±	11.22	71.36	±	12.47	62.42	±	11.31	64.17	±	6.92	68.13	±	6.81
ent-9A-12-*epi*-ST-Δ^10^-13-PhytoF	5.75	±	2.69	5.80	±	2.06	3.64	±	0.35	3.96	±	0.82	4.60	±	1.13	4.07	±	2.64
ent-16A-9-*epi*-ST-Δ^14^-10-PhytoF	6.95	±	0.75	5.83	±	1.47	6.60	±	0.59	6.81	±	1.53	8.42	±	1.76	5.48	±	3.50
ent-16B-9-*epi*-ST-Δ^14^-10-PhytoF	10.05	±	1.16	8.70	±	1.88	9.34	±	1.25	8.46	±	1.81	11.17	±	2.40	6.88	±	6.56
ent-16A-13-*epi*-ST-Δ^14^-9-PhytoF	9.09	±	11.95	4.95	±	5.86	1.03	±	0.27	0.68	±	0.11	0.83	±	0.16	1.50	±	1.15
ent-16B-13-*epi*-ST-Δ^14^-9-PhytoF	5.49	±	4.58	4.55	±	4.42	1.73	±	0.48	2.04	±	0.60	3.16	±	1.35	1.41	±	1.02
5(*R*)-5-F_3t_-lsoP	165.29	±	27.74	112.96	±	7.80	139.18	±	16.00	112.42	±	11.47	142.33	±	26.24	242.00	±	54.01
5(*S*)-5-F_3t_-lsoP	76.83	±	23.15	43.78	±	3.55	53.92	±	10.80	45.54	±	10.36	50.77	±	11.22	88.03	±	24.68
8(*R*)-8-F_3t_-lsoP	16.41	±	6.10	21.36	±	3.24	15.82	±	2.62	20.11	±	2.20	14.45	±	2.47	17.88	±	2.20
18(*R*)-8-F_3t_-IsoP	163.16	±	172.45	77.53	±	55.02	42.79	±	8.53	39.20	±	10.63	44.79	±	17.65	52.50	±	11.36
18(*S*)-8-F_3t_-IsoP	7.84	±	6.18	10.72	±	1.73	10.93	±	2.26	13.68	±	0.69	16.72	±	2.68	29.24	±	8.78

**Table 3:** Quantification of the nonenzymatic and enzymatic oxygenated metabolites in Mi168.

Mi168																					
Oxylipins	Control			0.25 mM of H_2_O_2_			1 mM of H_2_O_2_			2 mM of EtOAc			10 mΜ of EtOAc			29 g/L of NaCl			45 g/L of NaCl		
	Concentration (pg/mg)	±	SD	Concentration (pg/mg)	±	SD	Concentration (pg/mg)	±	SD	Concentration (pg/mg)	±	SD	Concentration (pg/mg)	±	SD	Concentration (pg/mg)	±	SD	Concentration (pg/mg)	±	SD
9-HODE	3.70 × 10^2^	±	3.27 × 10	4.39 × 10^2^	±	1.72 × 10^2^	2.49 × 10^2^	±	6.53 × 10	2.62 × 10^2^	±	4.07 × 10	4.22 × 10^2^	±	6.45 × 10	3.47 × 10^2^	±	7.87 × 10	2.70 × 10^2^	±	3.28
13-HODΕ	4.65 × 10^2^	±	4.61 × 10	5.21 × 10^2^	±	1.91 × 10^2^	3.22 × 10^2^	±	9.20 × 10	3.31 × 10^2^	±	6.70 × 10	5.20 × 10^2^	±	7.22 × 10	4.12 × 10^2^	±	7.05 × 10	3.58 × 10^2^	±	2.02 × 10
5-HΕΤΕ	3.66 × 10^3^	±	6.23 × 10	4.28 × 10^3^	±	1.70 × 10^3^	2.57 × 10^3^	±	5.18 × 10^2^	3.76 × 10^3^	±	3.71 × 10^2^	5.94 × 10^3^	±	2.41 × 10^3^	2.75 × 10^3^	±	5.15 × 10^2^	1.80 × 10^3^	±	1.67 × 10^2^
8-HΕΤΕ	2.23 × 10^3^	±	1.10 × 10^2^	2.73 × 10^3^	±	1.02 × 10^3^	1.57 × 10^3^	±	4.92 × 10^2^	2.19 × 10^3^	±	1.44 × 10^2^	3.26 × 10^3^	±	1.34 × 10^3^	1.67 × 10^3^	±	2.91 × 10^2^	8.63 × 10^2^	±	8.31 × 10
12-HΕΤΕ	3.07 × 10^3^	±	2.18 × 10^2^	3.67 × 10^3^	±	1.38 × 10^3^	2.18 × 10^3^	±	7.48 × 10^2^	2.80 × 10^3^	±	1.95 × 10^2^	4.42 × 10^3^	±	1.90 × 10^3^	2.27 × 10^3^	±	3.95 × 10^2^	1.29 × 10^3^	±	7.59 × 10
15-HΕΤΕ	4.22 × 10^3^	±	2.09 × 10^2^	5.06 × 10^3^	±	1.91 × 10^3^	2.95 × 10^3^	±	1.10 × 10^3^	3.88 × 10^3^	±	5.55 × 10^2^	6.04 × 10^3^	±	1.88 × 10^3^	3.33 × 10^3^	±	5.33 × 10^2^	2.48 × 10^3^	±	2.33 × 10^2^
5oxoETE	9.41 × 10^2^	±	6.88 × 10	1.19 × 10^3^	±	4.76 × 10^2^	6.73 × 10^2^	±	1.82 × 10^2^	9.59 × 10^2^	±	7.00 × 10	1.64 × 10^3^	±	6.79 × 10^2^	7.73 × 10^2^	±	1.20 × 10^2^	5.77 × 10^2^	±	7.91
8.9-EET	5.79 × 10^2^	±	3.04 × 10	6.05 × 10^2^	±	2.40 × 10^2^	3.92 × 10^2^	±	2.96 × 10	7.82 × 10^2^	±	2.11 × 10^2^	1.22 × 10^3^	±	5.95 × 10^2^	4.40 × 10^2^	±	5.47 × 10	3.12 × 10^2^	±	2.64 × 10
11.12-EET	4.37 × 10^2^	±	3.33 × 10	4.52 × 10^2^	±	1.36 × 10^2^	3.29 × 10^2^	±	4.50 × 10	6.02 × 10^2^	±	1.75 × 10^2^	9.73 × 10^2^	±	5.31 × 10^2^	3.52 × 10^2^	±	5.29 × 10	2.47 × 10^2^	±	2.58 × 10
14.15-ΕΕΤ	4.61 × 10^2^	±	5.58 × 10	4.98 × 10^22^	±	1.75 × 10^2^	3.24 × 10^2^	±	4.31 × 10	6.45 × 10^2^	±	1.52 × 10^2^	9.77 × 10^2^	±	5.55 × 10^2^	3.57 × 10^2^	±	5.98 × 10	2.11 × 10^2^	±	1.29 × 10
TXB2	3.86 × 10	±	1.14 × 10	4.47 × 10	±	3.08 × 10	2.65 × 10	±	6.16	2.92 × 10	±	8.55	7.86 × 10	±	2.82 × 10	2.88 × 10	±	4.45	1.31 × 10	±	4.00 × 10^−1^
PGD2	1.87 × 10	±	6.70	3.26 × 10	±	1.46 × 10	2.16 × 10	±	6.85	2.59 × 10	±	1.47	7.95 × 10	±	5.34 × 10	1.84 × 10	±	4.27	2.08 × 10	±	4.49
PGE2	1.57 × 10^2^	±	1.07 × 10^2^	8.41 × 10	±	6.39 × 10	1.05 × 10^2^	±	9.99 × 10	1.25 × 10^2^	±	7.83 × 10	1.17 × 10^2^	±	8.36 × 10	1.78 × 10^2^	±	1.24 × 10^2^	4.81 × 10	±	2.48 × 10
PGE3	6.59 × 10^2^	±	1.84 × 10^2^	8.48 × 10^2^	±	6.07 × 10^2^	3.80 × 10^2^	±	5.86 × 10	5.75 × 10^2^	±	1.24 × 10^2^	1.15 × 10^3^	±	5.73 × 10^2^	3.50 × 10^2^	±	5.02 × 10	2.10 × 10^2^	±	7.89
15-d-PGJ2	1.27 × 10	±	3.49 × 10^−1^	1.51 × 10	±	1.53 × 10	9.64	±	1.82	1.93 × 10	±	7.63	4.16 × 10	±	2.05 × 10	1.13 × 10	±	2.83	3.28	±	1.36
18-HEPE	4.71 × 10^4^	±	3.23 × 10^3^	5.87 × 10^4^	±	2.64 × 10^4^	3.41 × 10^4^	±	8.83 × 10^3^	4.24 × 10^4^	±	4.61 × 10^3^	7.26 × 10^4^	±	2.00 × 10^4^	4.70 × 10^4^	±	7.55 × 10^3^	3.37 × 10^4^	±	3.39 × 10^3^
RvD5	1.15 × 10	±	2.58	1.32 × 10	±	5.78	1.30 × 10	±	5.23	8.23	±	1.19	1.37 × 10	±	1.23 × 10	1.04 × 10	±	5.50	4.23	±	7.07 × 10^−2^
PDX	1.24 × 10	±	2.80	1.75 × 10	±	6.97	1.26 × 10	±	5.36	1.30 × 10	±	2.49	1.81 × 10	±	9.13	1.46 × 10	±	2.60	1.05 × 10	±	3.18
14-HDoHE	1.02 × 10^4^	±	1.15 × 10^3^	1.39 × 10^4^	±	5.14 × 10^3^	8.90 × 10^3^	±	2.15 × 10^3^	1.13 × 10^4^	±	1.19 × 10^3^	1.60 × 10^4^	±	6.11 × 10^3^	9.80 × 10^3^	±	2.28 × 10^3^	5.52 × 10^3^	±	8.59 × 10^2^
17-HDoHE	3.21 × 10^4^	±	4.23 × 10^3^	4.09 × 10^4^	±	1.37 × 10^4^	2.59 × 10^4^	±	1.09 × 10^4^	3.23 × 10^4^	±	4.76 × 10^3^	5.07 × 10^4^	±	1.20 × 10^4^	3.08 × 10^4^	±	5.16 × 10^3^	2.24 × 10^4^	±	3.87 × 10^3^
5(*RS*)-5-F_2c_-lsoP	111.13	±	3.01	115.61	±	4.08	90.74	±	20.99	90.61	±	2.74	129.47	±	15.19	100.71	±	33.99	133.33	±	21.74
5(*RS*)-5-F_2t_-lsoP	14.10	±	0.83	16.19	±	0.94	12.01	±	1.28	13.60	±	2.74	17.55	±	5.87	13.26	±	2.56	21.60	±	3.92
15-A_2_-IsoP	57.46	±	13.60	65.81	±	14.22	49.11	±	16.03	43.12	±	0.59	68.26	±	11.57	58.43	±	18.03	56.91	±	13.79
15-*epi*-15-F_2t_-IsoP	6.70	±	1.37	9.11	±	1.65	6.63	±	0.74	6.74	±	1.88	9.29	±	1.08	7.97	±	1.82	10.89	±	1.69
15(*RS*)-15-F_2t_-lsoP	3.33	±	0.26	4.42	±	1.79	3.64	±	0.42	3.38	±	0.56	5.07	±	1.87	3.32	±	0.41	5.13	±	0.55
4(*RS*)-14-F_3t_-NeuroP	18.59	±	2.37	23.44	±	4.62	19.18	±	3.00	17.33	±	1.10	22.13	±	5.16	19.76	±	4.11	33.50	±	4.55
9-L_1t_-PhytoP	3.09	±	0.74	6.22	±	3.77	1.22	±	0.25	4.29	±	2.57	2.95	±	0.87	2.24	±	0.56	3.56	±	0.99
16-B_1t_-PhytoP	3.71	±	0.55	6.98	±	4.56	1.34	±	0.33	4.51	±	2.98	3.82	±	1.04	2.99	±	0.20	4.69	±	1.08
16-*epi*-16-F_1t_-PhytoP	71.71	±	11.99	113.39	±	55.66	83.34	±	39.74	84.27	±	17.68	103.26	±	27.17	82.56	±	26.79	77.59	±	13.51
16-F_1t_-PhytoP	196.36	±	23.86	267.43	±	75.80	203.33	±	81.34	213.81	±	28.03	297.12	±	77.60	205.93	±	60.06	161.55	±	41.06
ent-16A-9-*epi*-ST-Δ^14^-10-PhytoF	1.60	±	0.37	3.17	±	1.55	1.02	±	0.18	1.52	±	1.08	2.09	±	0.39	1.49	±	0.18	2.00	±	0.05
ent-16B-9-*epi*-ST-Δ^14^-10-PhytoF	2.15	±	0.62	4.60	±	2.79	1.38	±	0.28	2.47	±	0.68	2.20	±	0.39	1.70	±	0.47	3.19	±	0.76
ent-16A-13-*epi*-ST-Δ^14^-9-PhytoF	1.77	±	1.16	2.24	±	2.14	0.67	±	0.15	0.57	±	0.41	1.22	±	0.56	0.98	±	0.11	1.02	±	0.73
5(*R*)-5-F_3t_-lsoP	247.40	±	45.11	283.17	±	43.02	209.98	±	31.07	204.07	±	37.03	319.24	±	64.80	279.09	±	64.13	495.36	±	9.54
5(*S*)-5-F_3t_-lsoP	87.74	±	12.50	96.90	±	21.94	74.47	±	6.85	76.78	±	18.69	109.24	±	31.62	99.86	±	26.60	171.49	±	5.36
8(*R*)-8-F_3t_-lsoP	13.05	±	3.25	18.33	±	5.29	12.24	±	2.03	13.59	±	1.22	25.82	±	6.01	15.82	±	2.63	25.63	±	4.18
8(*S*)-8-F_3t_-lsoP	12.07	±	3.39	11.00	±	3.69	8.44	±	1.99	8.65	±	2.56	12.66	±	2.99	10.87	±	2.76	11.96	±	2.89
18(*R*)-8-F_3t_-IsoP	392.01	±	375.37	659.01	±	738.83	83.54	±	19.69	89.87	±	42.52	167.32	±	43.50	159.82	±	34.50	199.22	±	27.56
18(*S*)-8-F_3t_-IsoP	23.03	±	5.23	27.85	±	1.37	20.46	±	3.16	20.45	±	10.01	27.20	±	6.80	28.07	±	4.57	51.69	±	5.57
4(*RS*)-4-F_4t_-NeuroP	8.23	±	0.49	10.72	±	2.18	8.12	±	1.46	7.84	±	0.24	10.36	±	2.27	8.44	±	2.00	13.65	±	1.38
10(*R*)-10-F_4t_-NeuroP	3.98	±	0.46	4.39	±	0.88	3.05	±	0.82	4.26	±	0.97	5.40	±	2.59	4.03	±	0.90	6.16	±	0.47
10(*S*)-10-F_4t_-NeuroP	3.17	±	0.59	4.22	±	2.26	3.52	±	0.84	2.87	±	0.29	4.57	±	1.39	4.71	±	0.86	5.06	±	0.32
13A(*RS*)-13-F_4t_-NeuroP	5.22	±	0.95	5.81	±	1.54	4.53	±	0.66	5.19	±	0.88	6.34	±	1.87	6.36	±	1.75	8.39	±	0.91
13B(*RS*)-13-F_4t_-NeuroP	4.28	±	0.61	5.92	±	0.34	4.39	±	0.58	2.68	±	1.99	3.82	±	0.36	3.95	±	0.63	5.46	±	1.94

## Data Availability

The data presented in this study are available on request from the corresponding author.

## Supplementary Materials

The following supporting information can be downloaded at: https://www.mdpi.com/article/10.3390/md22090406/s1, Table S1: Transitions (T1 and T2) in multiple reaction monitoring (MRM) mode for nonenzymatic oxylipins.
