# Supplementary material for: Stress-Induced Production of Bioactive Oxylipins in Marine Microalgae
Source: Mar Drugs. 2024 Sep 4;22(9):406. doi: 10.3390/md22090406 (PMC11432788; doi:10.3390/md22090406)
Supplement: Supplementary file 1 [file marinedrugs-22-00406-s001.zip › marinedrugs-3006978-supplementary.pdf]

Table S1: Transitions (T1 and T2) in multiple reaction monitoring (MRM) mode for non-enzymatic oxylipins

Analyte	m/z	MS2-T1	MS2-T2
ALA derivatives			
16-B_{1t}-PhytoP	307.2	235.0	223.0
9-F_{1t}-PhytoP	327.2	283.2	171.2
e*nt*-16-F_{1t}-PhytoP	327.2	283.2	251.2
9-L_{1t}-PhytoP	307.2	185.1	197.0
ent-16(*RS*)-13-*epi*-ST-D^{14}-9-PhytoF	343.2	201.0	127.0
ent-16(*RS*)-9-*epi*-ST-D^{14}-10-PhytoF	343.2	209.1	199.0
ent-9(*RS*)-12-*epi*-ST-D^{10}-13-PhytoF	343.2	237.1	86.9
ARA derivatives			
5-F_{2t}-IsoP	353.2	115	309
5-F_{2C}-IsoP	353.2	115	309
15-F_{2t}-IsoP	353.2	193	247
15-A2t-IsoP	333.2	271	189
EPA derivatives			
5-F_{3t}-IsoP	351.2	115	333
8-F_{3t}-IsoP	351.3	127.1	155
18-F_{3t}-IsoP	351.2	289.1	307.0
4-F_{3t}-NeuroP	379.2	101	299.3
14-F_{3t}-NeuroP	379.3	207	179
DHA derivatives			
4-F_{4t}-NeuroP	377.2	101	271.3
10-F_{4t}-NeuroP	377.2	153	110
13-F_{4t}-NeuroP	377.2	193	ND
